# Zinc status in HIV infected Ugandan children aged 1-5 years: a cross sectional baseline survey

**DOI:** 10.1186/1471-2431-10-68

**Published:** 2010-09-21

**Authors:** Grace Ndeezi, James K Tumwine, Bjørn J Bolann, Christopher M Ndugwa, Thorkild Tylleskär

**Affiliations:** 1 Department of Paediatrics and Child Health, School of Medicine, College of Health Sciences, Makerere University, Kampala, Uganda; 2 Centre for International Health, University of Bergen, Norway; 3 Institute of Medicine, University of Bergen, and Laboratory of Clinical Biochemistry, Haukeland University Hospital, Bergen, Norway

## Abstract

**Background:**

Low concentrations of serum zinc have been reported in HIV infected adults and are associated with disease progression and an increased risk of death. Few studies have been conducted in HIV infected children in Africa. We determined serum zinc levels and factors associated with zinc deficiency in HIV infected Ugandan children.

**Methods:**

We measured the baseline zinc status of 247 children aged 1-5 years enrolled in a randomised trial for multiple micronutrient supplementation at paediatric HIV clinics in Uganda (http://ClinicalTrials.gov NCT00122941). Zinc status was determined using inductively coupled atomic emission spectrophotometry (ICP-AES). Clinical and laboratory characteristics were compared among zinc deficient (zinc < 10.0 μmol/L) and non deficient children. Logistic regression was used to determine predictors of low serum zinc.

**Results:**

Of the 247 children, 134 (54.3%) had low serum zinc (< 10.0 μmol/L). Of the 44 children on highly active antiretroviral therapy (HAART), 13 (29.5%) had low zinc compared to 121/203 (59.6%) who were not on HAART. Overall, independent predictors of low zinc were fever (OR 2.2; 95%CI 1.1 - 4.6) and not taking HAART (OR 3.7; 95%CI 1.8 - 7.6).

**Conclusion:**

Almost two thirds of HAART naïve and a third of HAART treated HIV infected children were zinc deficient. Increased access to HAART among HIV infected children living in Uganda might reduce the prevalence of zinc deficiency.

## Background

Zinc deficiency is wide spread in low-income countries and is responsible for 4% of childhood deaths and 1% of the burden of disease in Africa, Latin America and Asia [1]. Populations in sub-Saharan Africa and South East Asia have the greatest risk of zinc deficiency because of inadequate zinc intake in about one third of the population [2].

Zinc is a component of various metallo-enzymes, proteins and cell membranes; and plays an important role in immune regulation. Zinc deficiency increases susceptibility to oxidative stress and impaired cell membrane function [3,4]. In HIV infected adults, low serum zinc has been associated with more advanced HIV disease and increased mortality [5-7].

Whereas some studies of HIV infected children in high-income countries have indicated that micronutrient deficiencies are uncommon [8], the reverse is true in low-income countries. The zinc status of HIV infected children in Uganda has not been reported. In this paper we report the magnitude of zinc deficiency and associated factors in a group of HIV infected children in Uganda.

## Methods

### Study sites

This study was part of a baseline assessment of children enrolled in a multiple micronutrient supplementation trial carried out between June 2005 and June 2008. This paper presents data from 3 of 7 clinics involved in the study, namely the paediatric HIV clinics at the national referral hospital (Mulago), Mildmay Centre and Nsambya hospital. These centres had laboratory facilities for blood sampling and freezing before transportation for analysis at a distant laboratory. Mulago is the national referral hospital; Mildmay Centre and Nsambya are private, not-for-profit hospitals. All the three are situated in the capital city, Kampala. The Mulago Hospital Paediatric HIV Clinic is the largest in the country and cares for more than 8000 patients. The Mildmay Centre, Uganda, is an HIV/AIDS referral and training institution, 12 kilometres South of Kampala city centre and cares for about 1500 HIV infected children, while Nsambya hospital cares for an equal number of HIV infected children.

### Design and subjects

We here report the baseline zinc levels of children aged 1-5 years who enrolled in the multiple micronutrient supplementation (MMS) study (ClinicalTrials.gov Identifier: NCT00122941).

The MMS study was a randomised trial of a 14 vitamins and minerals versus a six multivitamin supplement as 'standard-of-care' for 6 months, among 847 HIV infected children, with a highly active anti-retroviral therapy (HAART) strata comprising 10% of the study participants. The study enrolled HIV infected children who had at least attended the clinic once and were coming for follow-up. Those who were enrolled in other studies were excluded.

Children in the HAART strata had already been started on anti-retroviral therapy (ART) at the study clinics before enrolment into the study. The 2006 World Health Organization (WHO) guidelines for initiating anti-retroviral therapy in children had been used, in addition to a 'social criteria' that was conductive for initiating ART. Children aged 1 - 3 years were initiated on ART if their CD4+ T cell count was below 20% or if they had WHO stage 3 or 4 disease. Those above 3 years of age were initiated on ART if their CD4+ T cell count was < 15% or if they had WHO stage 3 or 4 disease[9]. The social criteria meant that the child had a consistent caretaker who was adherent to previous clinic appointments, attended at least 2 counseling sessions on adherence to ART and had consented for initiation of therapy.

**Figure 1:**
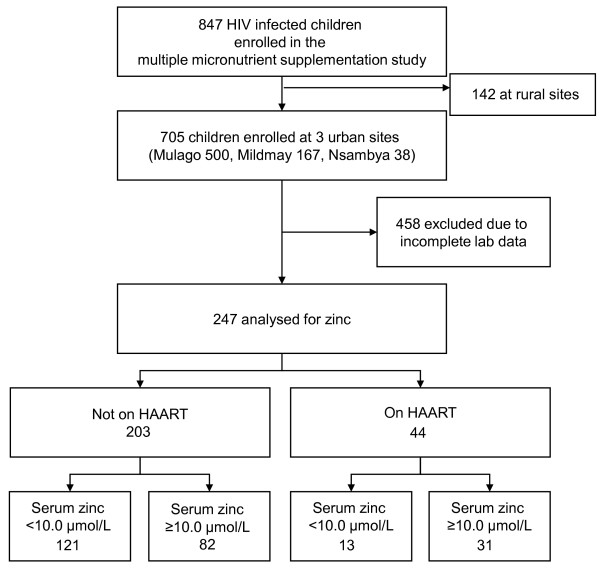
**Study profile**.

Due to cost, zinc was analysed at baseline only for children who had sufficient serum collected on both samplings (baseline and at 6-month follow-up). Of the 847 children who participated in the multiple micronutrient supplementation study, 705 were enrolled at the 3 sites where zinc analysis was possible. Two hundred and forty seven children of the 705 (35.0%) had complete clinical data and laboratory analyses at baseline to be included in this paper (figure 1). Laboratory data was declared incomplete as long as a participant did not have one or more of the tests. Out of the 458 exclusions, 261 children had haematology and CRP results but no micronutrient test (zinc inclusive) result, 64 had other micronutrient tests done except zinc, 89 had baseline zinc results but no second sample and 44 had missing samples. Overall insufficient samples contributed 414/458 exclusions while 44 were due to missing samples.

### Ethical issues

Written informed consent from the mothers/caretakers was obtained in English or Luganda, one of the commonly spoken local languages in the study area. Permission to conduct the study was granted by Makerere University College of Health Sciences, the Mildmay Centre, Nsambya and Mulago hospitals Research and Ethics Committees. Permission was also granted by the Uganda National Council for Science and Technology and the Regional Committee for Medical and Health Research Ethics, Western Norway ("REK Vest"). Counselling for initiation of HAART and adherence was offered to those who were eligible to start antiretroviral therapy. Treatment for illness and prophylaxis was offered according to the WHO and national paediatric HIV management guidelines.

### Procedures

#### History and physical examination

Mothers/caretakers were interviewed about the child's medical history, nutritional history and symptoms. A detailed physical examination was conducted by one of the study doctors. Weight was taken using a standardized uniscale 01-410-15 to the nearest 0.1 kg and height was taken using the Shorr portable infant/child length/height (Shorr productions, Olney, Maryland, USA) measuring board to the nearest 0.1 centimetre. HIV/AIDS clinical disease staging was determined using the World Health Organisation (WHO) classification for paediatric HIV/AIDS [10].

#### Laboratory procedures

Three to 5 millilitres (ml) of blood was collected in a 5 ml trace element free vacutainer tube (Becton Dickinson, Franklin Lakes, N.J) by venepuncture from the cubital fossa or dorsum of the hand irrespective of when the last meal was eaten. The sample was centrifuged at 2000 g within one hour of collection. Serum was transferred into trace element-free cryo-tubes, and transported within 3 hours to a minus 20°C refrigerator until shipment. Zinc was analysed using Inductively Coupled Atomic Emission Spectrophotometry (ICP-AES) [11] at the Clinical Chemistry Laboratory, Haukeland University Hospital, Bergen, Norway. The samples were digested using a microwave oven, nitric acid and concentrated hydrogen peroxide, as described by Rahil-Khazen et al [12]. The analytical coefficient of variation was about 3 %. An additional 2 ml sample was collected in an EDTA containing vacutainer tube and analysed for haemoglobin, white cell count and CD4+ T cell count within 12 hours of collection. A complete blood count was performed using the Act 5Diff instrument (Beckman Coulter) and CD4 count was done using a FACScan instrument and MultiSET software (Beckman Dickinson). Qualitative C-reactive protein (CRP) was measured using the latex immunoassay on one drop of serum (Human Gesellschaft fur Biochemica und Diagnostica mbH, Germany). Distinct agglutination indicated a C-reactive protein (CRP) content ≥ 6 mg/L. This rapid procedure was used in order to feedback the result to the attending paediatrician/doctor.

Low zinc was defined as a serum concentration below 10.0 μmol/L and an elevated CRP was defined as ≥ 6 mg/L. We used the WHO definition for anaemia where by children aged 6 months to 5 years are considered to be anaemic if their haemoglobin levels are below 11 g/dl [13]. We did not adjust the haemoglobin levels for ethnicity or altitude.

### Statistical issues

Data were analysed using SPSS version 15. Categorical characteristics were summarised into proportions while continuous variables were analysed using means and standard deviations. Demographic, anthropometry and clinical characteristics were compared among zinc deficient (zinc less than 10.0 μmol/L) and non deficient children using Fisher's exact test and odds ratios. All factors with a p-value of less than 0.20 by bivariate analysis were retained in the multiple logistic regression model to determine factors independently associated with low zinc. Weight for height (WHZ), height for age (HAZ) and weight for age (WAZ), z-scores were calculated using the WHO Anthro software and reference population [14] to assess anthropometric status.

## Results

Of the 247 children analysed for serum zinc, 134 (54.3%) had low zinc < 10μmol/L. Their mean (SD) age was 33.4 (13.8) with a range of 12.0 to 65.5 months. The male to female ratio was approximately 1:1.

### Clinical and haematological findings

Generally the children were unhealthy. More than half (141/247, 57.1%) had cough, which was the commonest symptom, followed by skin rash in about half (121/247, 49.0%) and fever in a fifth (94/247, 17.8%) of the children. More than half of the children (108/247, 53.0%) were stunted, almost a quarter were underweight (53/247, 21.5%) and a tenth was wasted. Twenty five percent (62/247) had advanced HIV disease with WHO stage 3 or 4. Absolute CD4+ T cell count ranged from 46 - 3769 with a mean (SD) of 1129(615). One third of the children (85/247) were severely immune-compromised with CD4+ T cell counts of less than 20%. Qualitative C-reactive protein was elevated in 100 out of 247 (40.5%) children. The mean haemoglobin (SD) was 9.9 (1.6) g/dl with a range of 4.6 - 13.7 g/dl. Two thirds of the children (170/247, 68.8%) were anaemic with haemoglobin of less than 11 g/dl.

There were no differences in sex, age, anthropometry, morbidity and CD4+ T cell count among those tested for zinc versus those who were not tested.

### Characteristics of children on HAART

The children receiving HAART were older with a mean (SD) age of 42.3 (10.7) compared to the non-HAART group with 31.4 (13.7) months. Other descriptive characteristics were similar in HAART treated and HAART naïve children as shown in table 1.

**Table 1:** Characteristics of HIV infected children aged 1-5 years by HAART status at paediatric HIV clinics in Uganda

Variable (mean, SD)	HAART (n = 44)	No HAART (n = 203)	Mean difference (95% CI)
Age in months	42.3 (10.7)	31.4 (13.7)	10.9 (6.6 - 15.2)
Weight for height z score	0.7 (1.4)	-0.2 (1.4)	0.9 (0.4 - 1.4)
Weight for age z score	-0.7 (1.1)	-1.3 (1.3)	0.6 (0.2 - 1.0)
Height for age z score	-2.1 (1.4)	-2.1 (1.6)	0.0 (-0.5 - 0.5)
Haemoglobin (g/dl)	10.9 (1.2)	9.8 (1.6)	1.1 (0.6 - 1.6)
Absolute CD4+ cell count (cells/μ L	1132 (664)	1128 (607)	6 (-198 - 206)
Serum zinc (μmol/L)	12.2 (4.1)	9.6 (2.5)	2.6 (1.6 - 3.5)

The proportion of HAART children who were tested for zinc (44/85, 55.0%) was significantly higher (p = 0.00) than the non-HAART (203/847, 32.5%) children analyzed for zinc. The mean serum zinc among children on HAART was 12.2 (SD 4.1) compared to 9.6 (SD 2.5) among those not receiving HAART, a statistically significant difference (OR 2.6; 95%CI 1.6 - 3.5). The mean duration of HAART was 10.2 months for the 13 zinc deficient and 9.5 months in children who were not zinc deficient, with a range of one to 21 months. Of the 44 children, 33 (75%) had received ART for 12 or more months.

### Serum zinc and factors associated with low zinc concentrations

**Figure 2:**
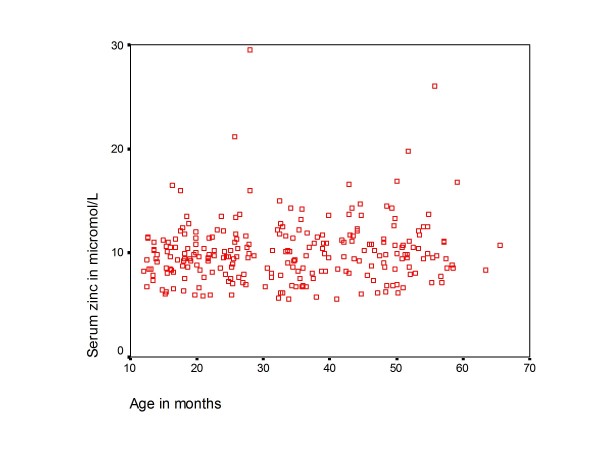
**A scatterplot of age against zinc levels**.

**Figure 3:**
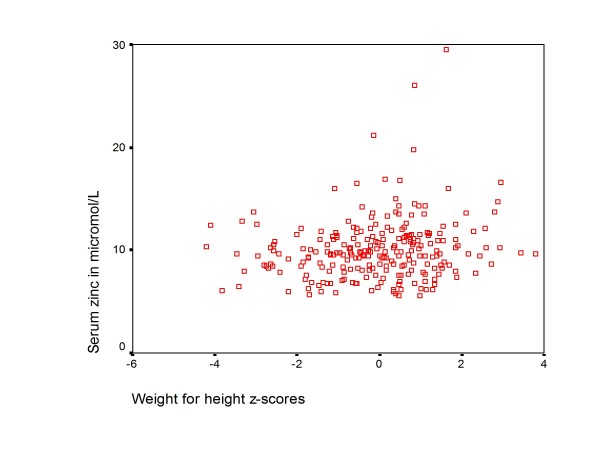
**A scatterplot of weight for height z-scores versus zinc levels**.

**Figure 4:**
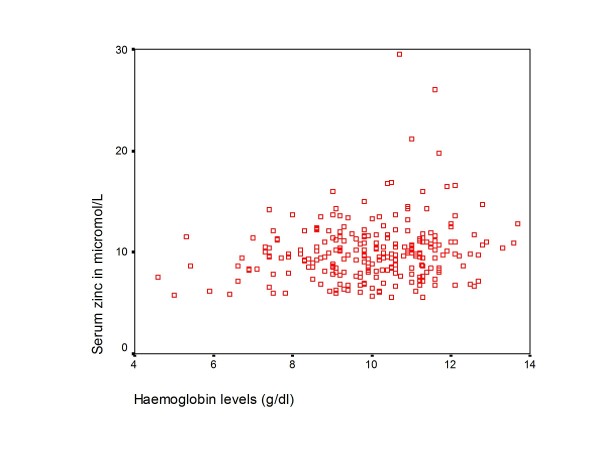
**A scatterplot of haemoglobin against zinc levels**.

The mean (SD) serum zinc concentration was 10.0 (2.9) μmol/L with a range of 5.6 - 29.5 μmol/L. There was no linear relationship between age, WHZ and haemoglobin versus zinc status as shown in Figure 2, 3 and 4. In addition there was no significant difference in mean age, anthropometry, haemoglobin and CD4+ cell count among children with low serum zinc (< 10μmol/L) compared to those with normal zinc levels (table 2).

**Table 2:** Characteristics of HIV infected children aged 1-5 years by zinc status at paediatric HIV clinics in Uganda

Variable (mean, SD)	Serum zinc < 10 μmol/L	95% CI	Serum zinc = 10 μmol/L	95% CI
Age in months	32.2 (14.0)	29.8 to 34.6	34.7 (13.6)	32.2 to 37.2
Weight for height z score	-0.2 (1.4)	-0.5 to 0.0	0.1 (1.5)	-0.2 to 0.4
Weight for age z score	-1.4 (1.3)	-1.6 to -1.2	-1.0 (-1.3)	-1.2 to -0.7
Height for age z score	-2.3 (1.4)	-2.5 to -2.0	-1.9 (1.8)	-2.3 to -1.6
Haemoglobin (g/dl)	9.7 (1.7)	9.5 to 10.0	10.2 (1.5)	9.7 to 10.5)
Absolute CD4+ cell count (cells/μ L)	1056 (613)	950 to 1162	1214 (610)	1099 to 1330
*On HAART (n,%)	13 (29.5)		31 (70.5)	

*Children on HAART are expressed as a percentage (%).

Factors associated with low serum zinc concentrations at bivariate analysis were: being HAART naïve, reported fever, underweight, WHO stage 3 or 4 disease and elevated CRP (table 3). The mean (SD) serum zinc among CRP negative children was 10.2 (2.5) compared to 9.8 (3.6) in CRP positive children. This difference was not statistically significant.

**Table 3:** Factors associated with low zinc in 247 HIV infected Ugandan children aged 1-5 years

Variable	No. of children	Low zinc < 10μmol/L (n; %)	Unadjusted OR (95%CI)	p-value	Adjusted OR (95%CI)
Age < 36 months Age ≥ 36 months	146 101	86 (58.2) 49 (48.5)	1.5 (0.9 - 2.5)	0.153	1.0 (0.6 - 1.8)
Male Female	128 119	75 (58.6) 59 (49.6)	1.4 (0.9 - 2.4)	0.162	0.7 (0.4 - 1.1)
On HAART No HAART	44 203	13 (29.5) 121(59.6)	3.5 (1.7 - 7.1)	0.000	3.7 (1.8 - 7.7)
Reported fever No fever	44 203	30 (68.2) 104 (51.2)	2.0 (1.0 - 4.1)	0.046	2.2 (1.1 - 4.6)
Current diarrhoea No Diarrhoea	20 227	15 (75.0) 119 (52.4)	2.7 (1.0 - 7.7)	0.062	2.2 (0.8 - 6.7)
Cough present No cough	141 106	81 (57.4) 53 (50.0)	1.4 (0.8 - 2.2)	0.249	
WHO stage 3 or 4 WHO stage 1 or 2	62 185	42 (67.7) 92 (49.7)	2.1 (1.2 - 3.9)	0.018	1.5 (0.8 - 2.9)
WHZ score < -2 WHZ score ≥ -2	24 223	15 (62.5) 119 (53.4)	1.5 (0.6 - 3.5)	0.519	
WAZ score < -2 WAZ score ≥ -2	56 191	38 (67.9) 96 (50.3)	2.1 (1.1 - 3.9)	0.022	1.2 (0.6 - 2.6)
HAZ score < -2 HAZ score ≥ -2	136 111	80 (58.8) 54 (48.6)	1.5 (0.9 - 2.5)	0.124	1.3 (0.8 - 2.3)
CRP < 6 mg/L CRP ≥ 6 mg/L	147 100	72 (49.0) 62 (62.0)	1.7 (1.0 - 2.9)	0.051	1.6 (0.9 - 2.7)
Hb < 11 g/dl Hb ≥ 11 g/dl	170 77	98 (57.6) 36 (46.8)	1.6 (0.9 - 2.7)	0.130	1.1 (0.6 - 2.0)
CD4 < 20% CD4 ≥ 20%	85 162	52 (61.2) 82 (50.6)	1.5 (0.9 - 2.5)	0.139	1.1 (0.6 - 1.9)

In the multivariate model the only significant independent predictors of low zinc were: being HAART naïve and reported fever. Of the 44 children on HAART, 13 (29.5%) had low serum zinc compared to 121 of 203 (59.6%) in the non-HAART group. This difference was statistically significant [Adjusted OR 3.7 (95% CI 1.8- 7.7)]. When fever was excluded from the model elevated CRP was a significant predictor of low zinc, illustrating that fever and CRP are closely related.

## Discussion

The prevalence of zinc deficiency in this group of HIV infected children aged 1-5 years attending paediatric HIV clinics in Uganda was high (54.3%). This was higher than what was reported in a previous study of children aged 6-36 months with persistent diarrhoea but of undetermined HIV status at Mulago hospital, Uganda [15]. While we used a cut off of 10 μmol/L to indicate zinc deficiency, the previous study had used 4.73 μmol/L assuming a normal serum zinc level of 8.99 (SD 2.13) μmol/L measured from a control group of healthy children. What was regarded as normal is below the mean (SD) serum zinc of 10.0 (2.9) reported in our study. An earlier community study in Kampala had found a serum zinc range of 8.4 - 20.9μmol/L [mean(SD) 10.1(3.2)] among children aged 4 -14 years[16]. Based on these two previous studies, it is possible that the zinc status in our study is similar to zinc levels in the general population of children in a similar age group in Uganda. The prevalence was twice as high in children who had not yet started HAART compared to those receiving HAART. This was not influenced by the duration on HAART. This implies that HAART may protect against zinc deficiency but will not completely eliminate it.

Compared to other studies of HIV infected children in Africa, the prevalence of zinc deficiency was higher than what was reported in South Africa and Rwanda [17,18]. Our study had a larger sample size and older children compared to the two studies which enrolled children from 2 months of age.

We did not find any association between age and zinc status in our study. All the children enrolled in this study were above one year of age, very few of whom were still breastfeeding. Other studies have shown that micronutrient deficiencies are less likely to occur below 24 months of age [17] and that breastfeeding is protective [19] against low serum zinc. Further more, there was no significant difference in mean zinc concentrations between girls and boys. Similar findings were reported from children whose HIV status was not known in low-income families in California [20].

Surprisingly, there was no significant association between zinc status and diarrhoea in the current study, possibly because there were very few children with diarrhoea. Studies in other developing countries have shown an association between low zinc and diarrhoea [21]. As expected, the presence of fever was significantly associated with low zinc levels. Fever is an indicator of infection and this may be associated with increased acute phase proteins and consequently reduced serum zinc [22]. Other common illnesses such as cough and skin rash were not associated with low zinc either.

There was a weak association between underweight and zinc status although a previous Uganda study of children with persistent diarrhoea [15] and a South Africa study of HIV infected children [17] did not find any association between zinc and nutritional status. Some authors have shown that severely malnourished children have a higher anti-oxidant activity and this is associated with low serum zinc [19]. Although anaemic children were more likely to have low zinc, the association was not significant. Other studies in zinc deficient low-income countries such as India and Cambodia have reported a close association between zinc deficiency and anaemia [19,23]. The lack of association in our study could not be explained.

Advanced HIV disease (WHO stage 3 and 4) was associated with low serum zinc but probably confounded by other factors. Advanced HIV disease is more likely to be associated with recurrent acute infections and an elevated acute phase response interpreted as low zinc. Our findings are similar to a South African study where the prevalence of zinc deficiency increased with HIV disease staging [6]. CD4+ T cell count had no association with zinc status in the current study. This finding is similar to what was reported in studies of HIV infected adults at Tufts university and Medical Centre in the nutrition for health living cohort [24] and in South Africa [6].

Children who were on HAART were less likely to be zinc deficient. This is further supported by a study in adults that showed that patients receiving HAART have better micronutrient indices including zinc than those not yet on HAART [25]. However, two other studies indicated that zinc deficiency remains highly prevalent in HIV infected adults on HAART [7,24].

We acknowledge that infant, child feeding practices and maternal HIV status influence the diet and mode of feeding [26], and may subsequently affect zinc intake. Adult studies have shown that the time of blood collection and feeding influence zinc levels[27], factors not controlled for in this study. The exclusions though numerous, were not systematic and therefore less likely to influence the results of our study. Low zinc levels were associated with fever or CRP implying that infection and acute phase proteins may have influenced the zinc status of the study children. Other researchers have previously confirmed that serum zinc is affected by the serum protein level and any acute-phase reactions[28]. Although the concentration of serum zinc gives limited information on the total zinc content in the body, there is no better way of determining zinc status that has been established[29]. The generalisability of this study is limited to the HIV infected children since we did not have a control group of HIV un-infected children.

Coupled with the already existing poor nutritional and immunological status, low zinc status in Ugandan HIV infected children is likely to remain a significant contributor to increased morbidity especially among those not yet receiving HAART.

## Conclusion

While almost two thirds of untreated HIV infected children were zinc deficient, zinc deficiency occurred in only a third of those on HAART. Increased access to HAART among HIV infected children living in Uganda might reduce the prevalence of zinc deficiency in this population.

## Competing interests

The authors declare that they have no competing interest.

## Authors' contributions

GN, TT and JKT participated in the conception, design and implementation of the study, statistical analysis, interpretation and drafting of the manuscript. CMN participated in study design and drafting of the manuscript. BJB analysed the serum samples for zinc and participated in drafting the manuscript. All authors read and approved the final manuscript.

## Pre-publication history

The pre-publication history for this paper can be accessed here:

http://www.biomedcentral.com/1471-2431/10/68/prepub
